# Effect of High-Dose Vitamin C on Tendon Cell Degeneration—An In Vitro Study

**DOI:** 10.3390/ijms252413358

**Published:** 2024-12-12

**Authors:** Shusuke Ueda, Toru Ichiseki, Miyako Shimasaki, Daisuke Soma, Masaru Sakurai, Ayumi Kaneuji, Norio Kawahara

**Affiliations:** 1 Department of Orthopaedic Surgery, Kanazawa Medical University, Daigaku 1-1, Uchinada-machi, Kahoku-gun 920-0293, Japan; 2 Division of Translational Research, Department of Life Science, Medical Research Institute, Kanazawa Medical University, Daigaku 1-1, Uchinada-machi, Kahoku-gun 920-0293, Japan; 3 Department of Pathology 2, Kanazawa Medical University, Daigaku 1-1, Uchinada-machi, Kahoku-gun 920-0293, Japan; miya0807@kanazawa-med.ac.jp; 4 Social and Environmental Medicine, Kanazawa Medical University, Daigaku 1-1, Uchinada-machi, Kahoku-gun 920-0293, Japan

**Keywords:** high-dose vitamin C, tendon cell degeneration, mitochondrial damage, oxidative injury, cellular senescence, actin filament

## Abstract

Tendinopathy is an aging-related disease, often caused by micro-scarring and degeneration due to overuse or trauma. Ascorbic acid (vitamin C) supplementation is reported to be a useful treatment for tendinopathy recovery. We compared the inhibitory effects of various ascorbic acid doses on tendon cell damage. H_2_O_2_ was added to human-derived tendon cells in vitro (Group H_2_O_2_, control), followed by incubation with 150 µM or 30 mM of ascorbic acid (Group C, Group HC). The oxidative injury degree was evaluated by determining reactive oxygen species levels. The cytoskeletal structure was examined via fluorescence immunostaining of actin filaments. Quantitative polymerase chain reaction (qPCR) was performed to analyze the expressions of mitochondria transcription factor A, adenosine triphosphate 5A, type I collagen, and p16. Cell death was reduced, and oxidative stress was inhibited in C and HC groups. The cytoskeleton was maintained in the HC group but not in the C group. qPCR analysis revealed that p16 expression was inhibited in both the C and HC groups compared to the H_2_O_2_ group; other markers had increased expression. The progression of cell death and cytoskeletal disruption was inhibited by the administration of high-dose vitamin C. Hence, high-dose vitamin C is a potential treatment for tendon cell degeneration.

## 1. Introduction

In young individuals, tendon damage occurs as a result of severe trauma, but in older adults, tendon ruptures and damage can occur even with minor trauma or activities of daily living. One of the reasons for this may be the aging and degenerative changes of tendons and ligaments. Tendon rupture and tendon damage associated with tendon degeneration and aging can occur in any part of the body, including the Achilles tendon, patellar tendon, and rotator cuff. When tendon damage or rupture occurs, it can cause inflammation in the surrounding tissue, potentially resulting in serious disruption to daily living. In recent years, the involvement of oxidative stress in degenerative tendinopathy has attracted attention, and its inhibition is considered important [1,2].

Recent reports have shown that oxidative stress in the Achilles tendon is involved in tendonitis and rupture and that oxidative stress is also related to rotator cuff injury and degenerative rotator cuff tears in the shoulder [3,4,5,6]. Additionally, there are conservative and surgical treatments for tendon rupture, but both carry the risk of re-rupture, which is also related to tendon tissue degeneration and aging-related disorders [7]. Therefore, it is believed that inhibiting tendon tissue degeneration is important for tissue repair and prevention of tendon damage.

As mentioned previously, oxidative stress has been reported to play a significant role in the degeneration and damage of tendon tissue. Furthermore, the involvement of advanced glycation end products (AGEs) or apoptosis in tendon injury has also been reported [7,8,9,10]. The accumulation of the end products of oxidation, such as AGEs, that accompanies tissue peroxidation and associated pathological conditions, may be improved by drugs with antioxidant properties.

Ascorbic acid (vitamin C) is a nutrient with strong antioxidant properties, and its effectiveness has been demonstrated in various fields [11,12,13]. The effectiveness of ascorbic acid in treating tendon injuries has also been reported [14,15,16]. However, recent reports have shown that although administration of ascorbic acid alone can suppress oxidative stress and the generation of AGEs, it is insufficient to suppress mitochondrial-mediated apoptosis [17,18,19]. Since the caspase activity pathway (intrinsic factor pathway) is involved in tendon cell degeneration, there may be limitations to its treatment or prevention with ascorbic acid.

The therapeutic effectiveness of high-dose vitamin C has been reported as a recent treatment method in various applications [20,21,22]. Specifically, high-dose vitamin C therapy inhibited the progression of nonalcoholic fatty liver disease [23]. Further, it has attracted attention due to its anti-aging effect [24]. Previous research has shown the inhibitory effects of ordinary-dose ascorbic acid on tendon cell degeneration; however, these effects were considered weaker than those elicited by other antioxidant agents. Therefore, this study compared the inhibitory effects of ordinary-dose versus high-dose ascorbic acid on tendon cell degeneration.

## 2. Results

### 2.1. Vitamin C Inhibits Reactive Oxygen Species (ROS) Production Due to Oxidative Stress

Tendon cells were exposed to 2 mM H_2_O_2_ and then cultured for 24 h after adding 150 µM, 7.5 mM, 15 mM, or 30 mM ascorbic acid. The ROS of the cultured cells was measured. In tendon cells exposed to H_2_O_2_ only, a significant increase in ROS levels was observed (*p* < 0.001). In the groups to which ascorbic acid was added, ROS was reduced in a concentration-dependent manner. In addition, a significant decrease in ROS was observed at 30 mM compared to 150 μM ascorbic acid (*p* < 0.05).

Thus, in this study, 2 mM H_2_O_2_ + 150 μM ascorbic acid (Group C) or 2 mM H_2_O_2_ + 30 mM ascorbic acid (Group HC) was added to the media of tendon cell cultures. H_2_O_2_(-), ascorbic acid(-), FBS(-) culture medium (normoxia group), and the medium (Group H_2_O_2_) to which 2mM H_2_O_2_ was added served as controls. These media were incubated for 3 h to detect ROS.

**Figure 1:**
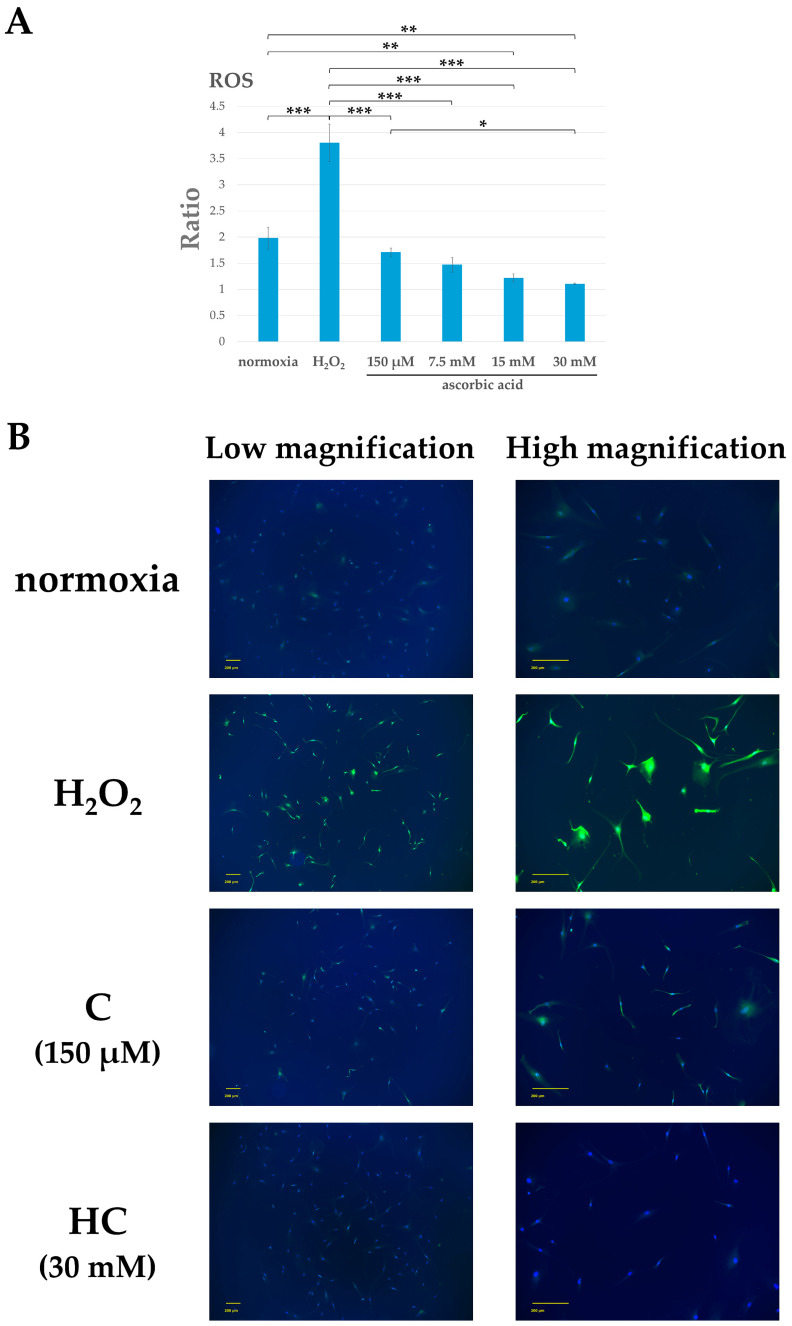
Reactive oxygen species (ROS) levels in tendon cells after H_2_O_2_ exposure. (**A**) ROS levels in tendon cells at 24 h after H_2_O_2_ exposure. Using a ROS Detection cell-based assay kit, ROS levels were measured in each group, as shown in the graphs (*n* = 5 each). Each graph indicates the ratio of ROS under the indicated conditions. Columns and bars indicate means and standard deviations, respectively. ROS levels increased with H_2_O_2_ exposure, but in the group to which ascorbic acid was added, ROS decreased in a concentration-dependent manner. There was a significant decrease in ROS at 30 mM compared to 150 μM ascorbic acid (* *p* < 0.05, ** *p* < 0.01, and *** *p* < 0.001). (**B**) ROS levels in tendon cells at 3 h after H_2_O_2_ exposure. The figure shows the H_2_O_2_, 2 mM H_2_O_2_ + 150 μM ascorbic acid (C), and 2 mM H_2_O_2_ + 30 mM ascorbic acid (HC) groups. Low- and high-magnification images are shown on the left and right rows, respectively. Representative fluorescence images for DAPI (blue) and ROS (green) are also shown. In the H_2_O_2_ group, expression of ROS was seen. Compared with the H_2_O_2_ group, ROS levels were reduced in both the C and HC groups. ROS was minimally detected in the HC group. Scale bar: 200 µm. DAPI, 4′,6-diamidino-2-phenylindole.

In the H_2_O_2_ group, ROS was detected in many cells. ROS production was inhibited in the two groups treated with ascorbic acid. A noticeable inhibitory effect on ROS levels was found in the HC group (Figure 1).

### 2.2. Vitamin C Protects Against Cytoskeletal Damage in Tendon Cells Due to Oxidative Stress

**Figure 2:**
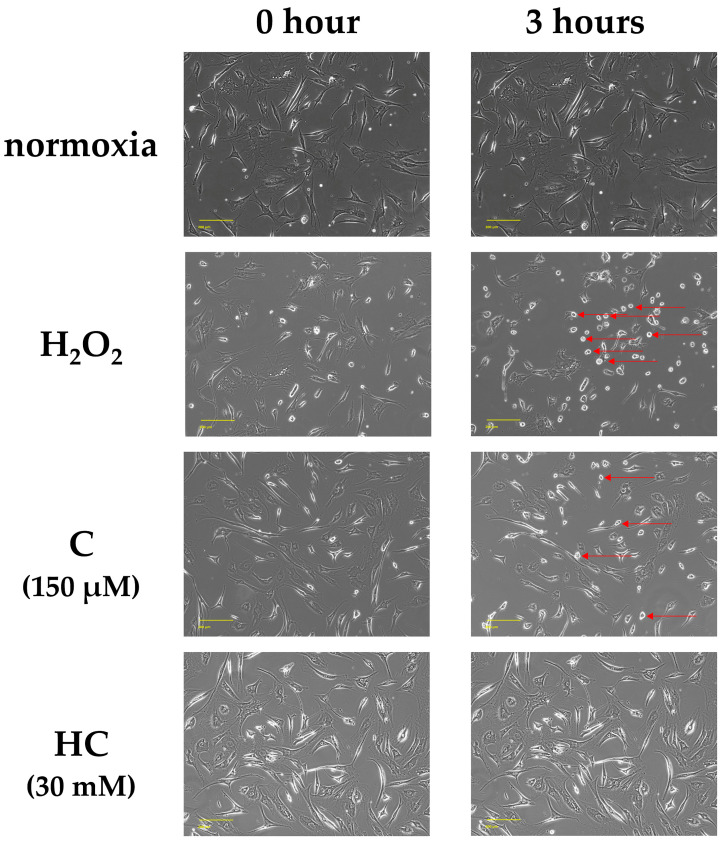
Phase-contrast images of tendon cells at 3 h after H_2_O_2_ exposure. The figures show representative images of various treatment groups. In the normoxia group, most of the cells were engrafted, and almost no detached cells were observed. The H_2_O_2_ group showed a large number of round or shrunken cells. The round and shrunken cells were released, and cell death was observed (red arrows). In the C group, cells with round and shrunken nuclei (red arrows) were mixed with cells that maintained their original forms. Compared with the H_2_O_2_ group, the number of dead cells was reduced in both the C and HC groups. In the HC group, the number of cell deaths was reduced compared to that in the C group. Scale bar: 200 µm.

After adding the respective treatments, the cell morphology was compared across the four groups. After 3 h of incubation, the H_2_O_2_ group showed a large number of round or shrunken cells that appeared to be dead [25,26]. In the C group, cells with round and shrunken nuclei were mixed with cells that maintained their original forms. In the HC group, the cell form was preserved to the same degree as that of the normoxia group (Figure 2).

**Figure 3:**
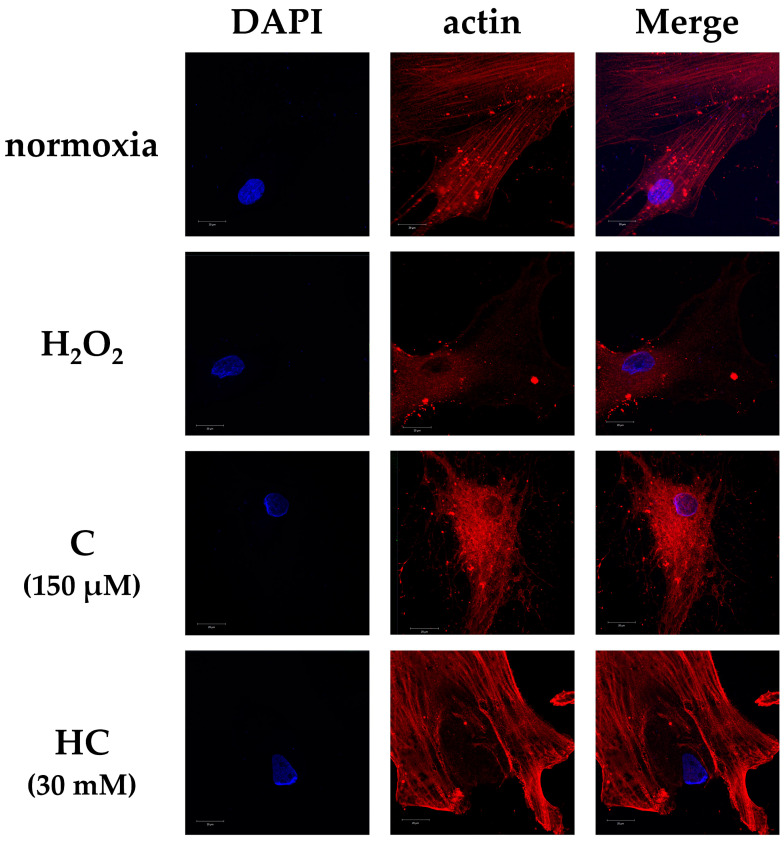
Fluorescence immunostaining of actin filaments in tendon cells at 3 h after H_2_O_2_ exposure. The figure shows representative fluorescence images for DAPI (blue) and/or actin (red) in the various treatment groups. Actin filaments had disappeared in the H_2_O_2_ and C groups. In the HC group, actin filaments were preserved to the same degree as those of the normoxia group. Scale bar: 20 µm.

Fluorescence immunostaining showed a definite disappearance of actin filaments in the H_2_O_2_ group. In the normoxia group, actin filaments were detected, and the appearance of the cytoskeleton was maintained. However, in the C group, the actin filaments had disappeared. In the HC group, the actin filaments and the cytoskeleton were maintained to the same degree as those of the normoxia group (Figure 3).

### 2.3. Vitamin C Maintains Mitochondrial Function, Energy Production, and the Extracellular Matrix from Cellular Damage Due to Oxidative Stress and Inhibits the Expression of Cellular Senescence-Related Genes

**Figure 4:**
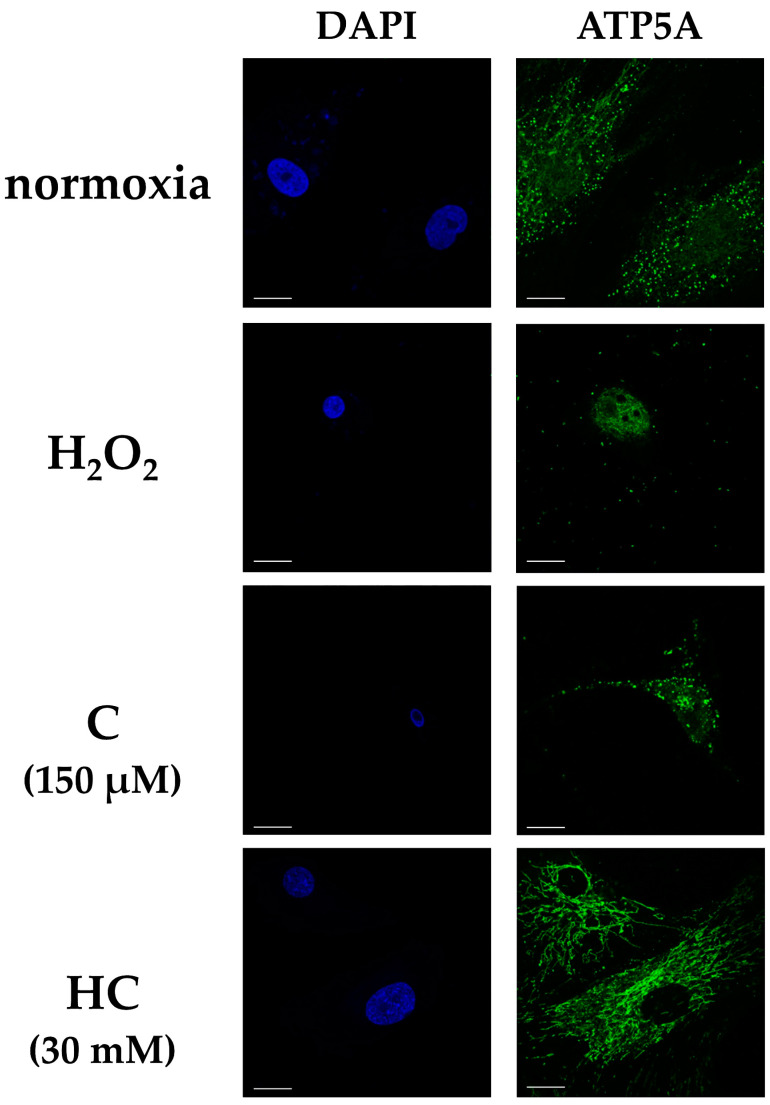
Fluorescence immunostaining of ATP5A in tendon cells at 24 h after H_2_O_2_ exposure. The figure shows representative fluorescence images for DAPI (blue) or ATP5A (green) in the various treatment groups. In the HC group, expression of ATP5A was seen. Compared with the HC group, ATP5A was reduced in both the H_2_O_2_ and C groups. Scale bar: 20 µm. ATP5A, adenosine triphosphate 5A.

Each group was cultured for 24 h, and adenosine triphosphate 5A (ATP5A) was compared using immunofluorescence staining. ATP5A expression was observed in all the groups, but the expression was decreased in the H_2_O_2_ group compared with the other groups. The HC group showed increased expression of ATP5A compared with the H_2_O_2_ and C groups. Shrinkage of the nucleus was observed in the H_2_O_2_ and C groups (Figure 4).

**Figure 5:**
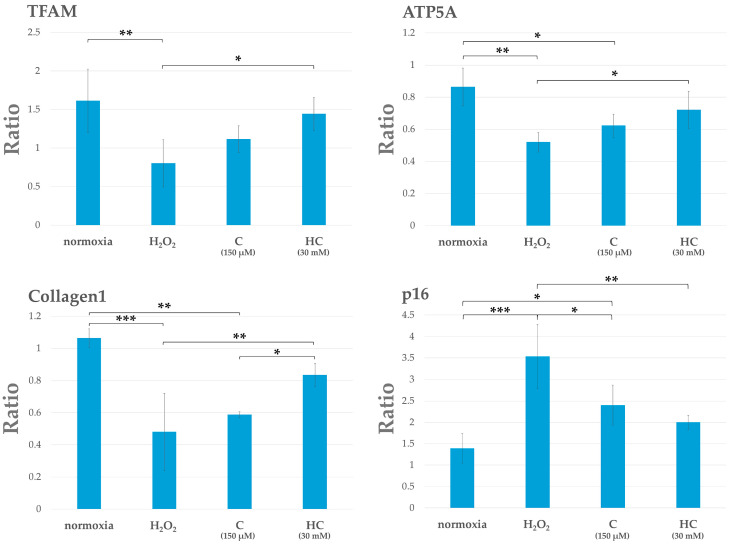
qPCR analysis of gene expression in tendon cells at 3 h after H_2_O_2_ exposure. Quantitative RT-PCR analysis of each gene expression level was conducted in each group, as shown in the graphs (*n* = 3 each). Each graph indicates the ratio of each gene expression level under the indicated conditions. Columns and bars indicate means and standard deviations, respectively. The H_2_O_2_ group showed a decrease in the expression levels of TFAM, ATP5A, and type I collagen and an increase in the p16 expression level. In the C and HC groups, an increase in the expression levels of TFAM, ATP5A, and type I collagen was seen as compared with the H_2_O_2_ group, although the expression level of p16 decreased. This was particularly noticeable in the HC group compared to the C group (* *p* < 0.05, ** *p* < 0.01, and *** *p* < 0.001). qPCR, quantitative polymerase chain reaction; RT-PCR, reverse transcription polymerase chain reaction.

The expressions of the mitochondrial markers are as follows: mitochondria transcription factors A (TFAM) and ATP5A, the extracellular matrix synthetic protein type I collagen, and the senescence factor p16: CDKN2A (cyclin-dependent kinase inhibitor 2A). These were analyzed via quantitative polymerase chain reaction (qPCR). Each group was incubated for 3 h. The results showed that the expressions of TFAM, ATP5A, and type I collagen decreased in the H_2_O_2_ group compared to the normoxia group. In the C and HC groups, the expressions of these genes increased compared to the H_2_O_2_ group; gene expression was higher in the HC group than in the C group. The level of p16 expression was significantly higher in the H_2_O_2_ group than in the other groups. However, p16 expression was inhibited in both the C and HC groups; it was particularly noticeable in the HC group (* *p* < 0.05, ** *p* < 0.01, and *** *p* < 0.001) (Figure 5).

### 2.4. High-Dose Vitamin C Maintains Cell Viability by Protecting the Cytoskeleton from Cell Damage in Tendon Cells Previously Exposed to Oxidative Stress

**Figure 6:**
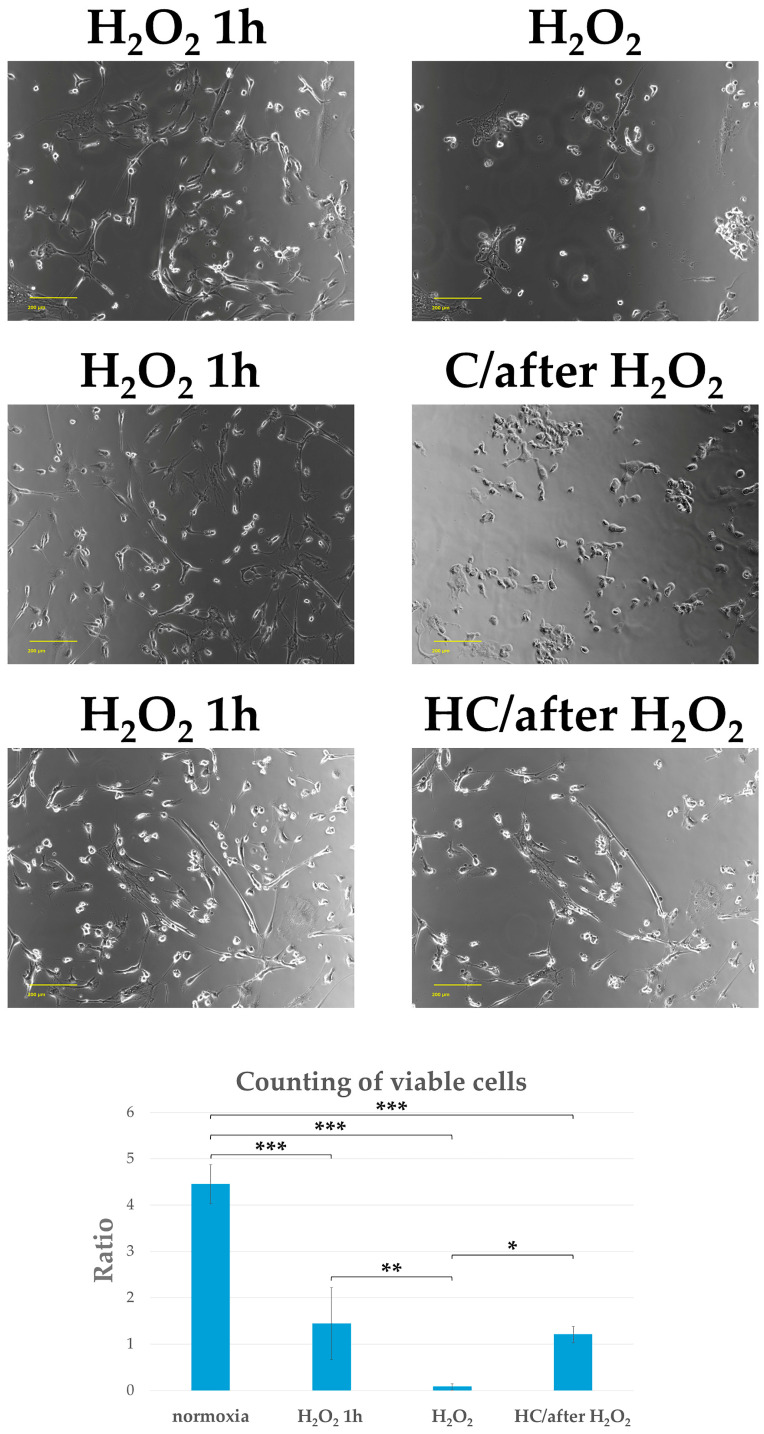
Phase-contrast image of tendon cells treated with 150 µM or 30 mM ascorbic acid added 1 h after H_2_O_2_ exposure. Shown are the representative phase-contrast images of the cells in each group at 1 h after H_2_O_2_ exposure and then at 2 h after adding 150 µM or 30 mM ascorbic acid (C/after H_2_O_2_ group and HC/after H_2_O_2_ group). The numbers of viable cells were measured in each group, as shown in the graphs (*n* = 3 each). Each graph indicates the ratio of viable cells under the indicated conditions. Columns and bars indicate means and standard deviations, respectively. One hour after exposure to H_2_O_2_ (H_2_O_2_ 1 h group), round and shrunken cells were seen together with mature tendon cells. The number of adherent cells was reduced in the H_2_O_2_ group. Contrastingly, the number of adherent cells in the HC/after H_2_O_2_ group was maintained. The number of adherent cells in the C/after H_2_O_2_ group was reduced to the same degree as in the H_2_O_2_ group. The number of viable cells was significantly maintained in the HC/after H_2_O_2_ group compared to the H_2_O_2_ group (* *p* < 0.05, ** *p* < 0.01, and *** *p* < 0.001). Scale bar: 200 µm.

**Figure 7:**
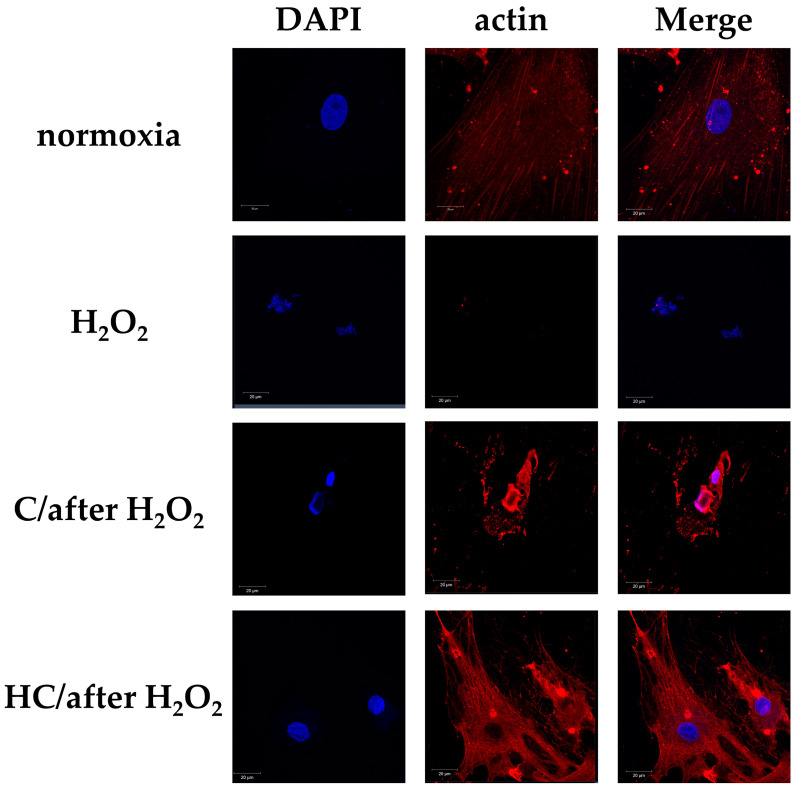
Fluorescent immunostaining of tendon cell actin filaments exposed to 150 µM or 30 mM ascorbic acid at 1 h after H_2_O_2_ exposure. Shown are representative fluorescence microscopy images for DAPI (blue) and/or actin (red) in the cells of all treatment groups. In the H_2_O_2_ group, a noticeable destruction in cell form and a loss of nuclear and actin filaments were observed. Contrastingly, the HC/after H_2_O_2_ group maintained the cell form, cytoskeleton, and actin filament expression. The C/after H_2_O_2_ group showed approximately the same degree of cell form destruction and actin filament loss as the H_2_O_2_ group. Scale bar: 20 µm.

In this experiment, 150 µM or 30 mM ascorbic acid was added to the tendon cell culture medium that had been incubated for 1 h with H_2_O_2_ (C/after H_2_O_2_ group and HC/after H_2_O_2_ group). We then determined the cell morphology, viable cell count, and the presence of actin filaments after 2 h. One hour after exposure to H_2_O_2_ (H_2_O_2_ 1 h group), round and shrunken cells were seen together with mature tendon cells. Two hours after adding a high dose of ascorbic acid, the cell form and presence of actin filaments were observed. The H_2_O_2_ group showed clear disruptions in nuclear and cellular morphology, a decrease in adherent cell count, and a marked loss of actin filaments. However, in the HC/after H_2_O_2_ group, the adherent cell count and cell form, actin filaments, and cytoskeleton were maintained. The C/after H_2_O_2_ group showed cell morphological destruction and a reduction in adherent cell number, as well as a loss of actin filaments, similar to the H_2_O_2_ group. The viable cell count was significantly maintained at 2 h after adding a high dose of ascorbic acid to the tendon cell culture medium that had been incubated with H_2_O_2_ for 1 h (HC/after H_2_O_2_ group), compared with the H_2_O_2_ group (* *p* < 0.05, ** *p* < 0.01, and *** *p* < 0.001) (Figure 6 and Figure 7).

## 3. Discussion

This study examined the prevention of tendon cell degeneration and its treatment. Inhibiting oxidative stress or mitochondrial damage at an early stage is important in suppressing the progression of tendon cell degeneration [19]. Thus, we studied the responses of the tendon cells in the early stages after H_2_O_2_ exposure. Our results showed that oxidative injury was significantly inhibited with high-dose vitamin C compared to ordinary-dose vitamin C, in addition to the maintenance of the tendon cells themselves.

The cyclin-dependent kinase (CDK) inhibitor and cellular senescence marker p16^INK4a^ inhibit CDK4/6, reducing the phosphorylation of Rb protein, which is necessary for progression through the DNA replication phase of the cell cycle and for causing the cell cycle to stop [27,28]. Additionally, the removal of p16^INK4a^-positive cells has been reported to improve various age-related pathologies [29]. Since p16^INK4a^ also affects regeneration and repair in cardiac muscle cells, it regulates the promotion of cardiac muscle cell proliferation via CDK4/6 and ROS-related autophagy. Additionally, overexpression of p16^INK4a^ overactivates autophagy, increasing myocardial damage and weakening myocardial repair [30]. In this study, an increase in p16^INK4a^ expression was observed in tendon cells exposed to H_2_O_2_; however, treatment with high-dose vitamin C inhibited p16^INK4a^ expression. This finding demonstrated that high-dose vitamin C administration is effective against muscle and tendon cell damage.

Mitochondria produce adenosine triphosphate (ATP), a molecule necessary for life activity, contributing to the maintenance of cell functions and reasonable stress responses. When mitochondrial dysfunction occurs, ATP production is reduced, and ROS production, a cause of DNA damage, is increased. This reduces cellular function, causes cellular transformation, and induces cellular senescence, cell death, or inflammation, finally leading to damage to body tissue or organ function [31,32,33,34]. Tendinopathy is an aging-related disease, and degeneration is said to be the cause. Degenerated tendons are a significant source of ROS. Recent studies have reported that mitochondrial dysfunction is involved in tendinopathy [35]. The presence of TFAM is responsible for maintaining and stabilizing mitochondrial DNA. Since mitochondrial DNA is easily subjected to oxidative injury, PCR-based gene expression analysis was performed to investigate the effects of oxidative injury on mitochondria in tendon cells where degeneration is induced. Both TFAM expression and mitochondrial function were reduced in degenerated tendon cells. However, treatment with high-dose vitamin C increased TFAM expression and maintained mitochondrial function. These results reveal that high-dose vitamin C protects mitochondria, inhibiting oxidative injury and tendon cell degeneration. Furthermore, oxidative stress causes the oxidation of actin, which promotes the formation of stress fibers and oxidizes actin regulatory proteins, resulting in the impairment of normal actin polymerization [36]. As mitochondria interact with the actin cytoskeleton, mutations in actin or actin-binding proteins affect the mitochondrial pathway and lead to cell death [37]. In this experiment, high-dose vitamin C suppressed actin oxidation by reducing the levels of ROS induced by H_2_O_2_. Moreover, it is believed that by protecting mitochondria, the actin cytoskeleton was maintained, and cell death was suppressed. These results also demonstrated that treating tendon cells with high-dose vitamin C maintains cytoskeletal morphology, raising the possibility that high-dose vitamin C is effective for inhibiting the progression of tendon cell degeneration. Collectively, the results of this study suggested that mitochondrial damage and senescence factors are involved in the development of tendon cell degeneration and injury. Therefore, mitochondria should be protected and the degeneration of tendon cells should be inhibited by suppressing cellular senescence. In this study, treatment with high-dose vitamin C maintained the cytoskeleton and increased the gene expression of type I collagen, suggesting a decrease in the risk of degenerated tendon injury.

Restoring a degenerated tendon tissue to a healthy tendon tissue is currently impossible. Only a few studies have addressed the therapeutic effectiveness or inhibitory effects for degenerative tendinopathy. It is important to inhibit mitochondrial damage and oxidative stress at an early stage to prevent mitochondria-mediated apoptosis and to inhibit tendon cell degeneration. Therefore, we investigated the therapeutic effect of vitamin C after H_2_O_2_ exposure and the occurrence of tendon cell degeneration; an increase in dead cells was observed 1 h after H_2_O_2_ exposure, followed by a high dose of vitamin C, which considerably suppressed cell death. Additionally, the cytoskeleton was maintained when high-dose vitamin C was added 1 h after H_2_O_2_ exposure. In other words, treatment of tendon cells with high-dose vitamin C showed promising results in inhibiting the progression of their degeneration. These results suggest that mitochondrial damage and senescent cells may be suppressed even when tendon cells are degenerated, and high-dose vitamin C may be expected to have a favorable postoperative effect even in cases where tendon tissue is degenerated and at risk of re-rupture.

Limitations of this study are as follows: (1) the study was conducted in vitro (not in vivo), and (2) the study was based on the occurrence of tendon cell death caused by H_2_O_2_; hence, it was conducted for a maximum of 24 h, and the effects of ascorbic acid on long-term tendon degeneration and tendon damage are not known. Although we believe it is necessary to conduct further studies in animal experiments, this study demonstrates the efficacy of high concentrations of ascorbic acid on tendon cells; these findings provide a valuable foundation for future studies. (3) In this study, we examined TFAM with a focus on mitochondrial function, but we did not examine Ca flux, so more detailed studies on the mechanisms of mitochondrial damage and cell death may be needed [38,39].

Ascorbic acid is an inexpensive and clinically used drug. Although the safety of high-dose administration of vitamin C has been reported [23,40], there are some caveats. Gastrointestinal discomfort and kidney stones are possible when large doses of vitamin C are administered. Caution should be applied when administering high doses of vitamin C, especially in patients suffering from kidney disease or hemochromatosis [40,41]. It is also reported that 40 mM ascorbic acid can be considered as the minimal concentration to induce significant cell changes, and thus the toxicological threshold and the concentration should be carefully considered when used [42]. Therefore, its use should be administered under the supervision of a health care provider or health care professional. Considering the results of this study and the safety concerns reported thus far, high-dose vitamin C therapy, which should be carefully monitored with careful monitoring of physical condition, may be an effective prevention and treatment for a class of injuries resulting from degeneration of tendons, including the Achilles tendon, patellar tendon, and shoulder rotator cuff.

## 4. Materials and Methods

### 4.1. Cell Culture

Human tendon cells (Zen-bio, Durham, NC, USA) were seeded into culture dishes and incubated for 24 h in a humidified incubator at 37 °C with 95% air and 5% CO_2_ to allow for cell attachment and proliferation. Tendon cells were maintained as subconfluent monolayer cultures in a tenocyte growth medium (Zen-bio) supplemented with 10% fetal bovine serum (FBS; GIBCO-BRL, Grand Island, NY, USA) at 37 °C under 20% O_2_ and 5% CO_2_. When the cultures reached 70% confluence, 2 mM H_2_O_2_ (WAKO, Tokyo, Japan) + 150 μM ascorbic acid (WAKO) (Group C) or 2 mM H_2_O_2_ + 30 mM ascorbic acid (WAKO) (Group HC) was added to the medium, and the cells were incubated for 3 h. In the next experiment, 150 µM or 30 mM ascorbic acid was added to the tendon cell culture medium that had been incubated for 1 h with H_2_O_2_ (C/after H_2_O_2_ group, HC/after H_2_O_2_ group). The cells were incubated for a total of 3 h and then cultured under 20% O_2_ in a medium without H_2_O_2_, ascorbic acid, or FBS (GIBCO-BRL; normoxia group) or in a medium containing H_2_O_2_ only (Group H_2_O_2_). Three independent experiments were performed for each group.

### 4.2. Analysis of Reactive Oxygen Species (ROS)

ROS was determined using a commercial DCFDA-cellular ROS detection assay kit following the manufacturer’s instructions (Cayman Chemical Company, Ann Arbor, MI, USA). Briefly, tenocyte was plated on a 96-well plate (2.5 × 10^4^ cells/well). After overnight attachment, the cells were treated with H_2_O_2_ at a designated concentration for 24 h, followed by staining with a cell permeant reagent, 2′,7′-dichlorofluorescein diacetate (DCFDA), for 30 min at 37 °C. Deacetylated DCFDA was fluorescently determined after being oxidized by ROS with Ex 495 nm/Em 529 nm by fluorescence microplates (Fluoroskan, Thermo Fisher Scientific, Waltham, MA, USA). In each group, 5 wells were assayed 3 times.

### 4.3. Detection of Intracellular ROS Generation

The experiments were conducted in accordance with the protocol provided with the ROS Assay Kit-Photo-oxidation Resistant DCFH-DA (Dojindo, Kumamoto, Japan). ROS production was analyzed using the ROS Assay Kit-Photo-oxidation Resistant DCFH-DA. Cells were seeded on dishes or plates and placed in an incubator at 37 °C with 95% air and 5% CO_2_. The culture medium was then removed, and the cells were washed twice with Hank’s basic salt solution (HBSS). An appropriate volume of photo-oxidation resistant DCFH-DA working solution was then added to the cells. The cells were then incubated as in step 1 for 30 min. The working solution was then discarded, and the cells were washed with HBSS. Next, a medium containing a ROS-inducing agent was then added, and the cells were incubated as in step 1 for an appropriate time. After removing the supernatant, the cells were washed twice with phosphate-buffered saline (PBS). The supernatant was then replaced with DAPI (1 μg/mL) in a blocking solution, and the cells were incubated at room temperature for 1 h. Finally, fluorescence microscopy (470 nm and 530 nm LED modules) was performed using a BZ-X700 fluorescence microscope (Keyence, Tokyo, Japan).

### 4.4. Quantitative RT-PCR Analysis

Total RNA was isolated according to an RNA extraction kit (Isogen, Nippon Gene, Tokyo, Japan), which included a DNase digestion step. cDNA was synthesized using a High-Capacity RNA-to-cDNA Kit (Thermo Fisher Scientific) in a mixture containing 1 μg of total RNA in 20 μL. Real-time qPCR analysis was performed on cDNA equivalent to 20 ng RNA in a 10 μL reaction volume using a QuantStudio 3 Real-Time PCR system (Thermo Fisher Scientific). Gene expression analysis in human tendon cells was measured using a TaqMan Fast Advanced Master Mix (Thermo Fisher Scientific) and one of the following TaqMan gene expression assays (Thermo Fisher Scientific): TFAM (Hs01082775_ml), ATP5A (Hs00900735_ml), CDKN2A (Hs00923894_ml), Collagen1 (Hs00164004_ml). It was then normalized to 18srRNA (Hs03003631_g1). The threshold cycle (Ct) was determined after setting the threshold in the linear amplification step of the PCR reaction. The ΔCt of a specific gene was defined as the Ct (target gene) − Ct (18srRNA). Data were analyzed using the relative quantification (ΔΔCt) method in QuantStudio Design & Analysis software v1.5 (Thermo Fisher Scientific). The data are shown as averages of three independent experiments.

### 4.5. Immunostaining for F-Actin and ATP Synthase (ATP5A)

The cultured cells were fixed in 4% paraformaldehyde, washed in PBS, and permeabilized with 0.3% Triton X-100 in PBS. Nonspecific binding was blocked by incubating the cells with 10% bovine serum albumin (Dako Cytomation, Santa Clara, CA, USA) in PBS for 15 min. They were then incubated with anti-actin antibodies (1:200; Sigma Aldrich, St. Louis, MO, USA) and anti-ATP5A antibodies (1:200; Proteintech, Rosemont, IL, USA) for 2 h. The cells were then incubated with Alexa 594-labeled secondary antibodies (Thermo Fisher Scientific, Waltham, MA, USA) for anti-actin and Alexa 488-labeled secondary antibodies (Thermo Fisher Scientific) for anti-ATP5A. The cells were then incubated with 4′,6-diamidino-2-phenylindole (DAPI) for 30 min. After washing, a Prolong diamond antifade mountant (Thermo Fisher Scientific) was added, and coverslips were mounted. Finally, fluorescence microscopy images were taken using a Zeiss-LSM710 microscope (Zeiss, Baden-Württemberg, Germany).

### 4.6. Determination of Cell Viability

The tendon cells were cultured at a density of 5.0 × 10^4^ cells/well in a 24-well plate. One group comprised tendon cells exposed to 2 mM H_2_O_2_ for 1 h (H_2_O_2_ 1 h group) before adding 30 mM ascorbic acid. The cells were then incubated in the tendon cell culture medium containing 30 mM ascorbic acid for 2 h (HC/after H_2_O_2_ group). The cells were incubated for a total of 3 h. Another group was exposed to 2 mM H_2_O_2_ only for 3 h (H_2_O_2_ group). After exposure to the respective treatments, the cells were stained using the Trypan blue (0.25%) dye exclusion method, the numbers of viable and nonviable cells were determined using a Countess 2 FL instrument (Thermo Fisher Scientific), and the survival rates were calculated. The control cells (normoxia group) were cultured in a medium without H_2_O_2_, ascorbic acid, or FBS (GIBCO-BRL). The tendon cells were cultured in three wells for all groups. In each group, the three wells were measured three times.

### 4.7. Statistical Analysis

All quantified results were expressed as the mean ± deviation (SD). Statistical significance was analyzed using a one-way analysis of variance followed by Fisher’s protected least significant difference post-hoc test. Significance was defined at *p*-values < 0.05. Statistical analyses were performed using Stat View J-5.0 software (SAS Institute, Cary, NC, USA).

## 5. Conclusions

Although the tendon cell lines used in this study were in vitro studies derived from human Achilles and patellar tendons, the results of this study suggest that the addition of high-dose vitamin C, which has a strong antioxidant effect, may play a protective role in tendon cells.

This study may help in the prevention and treatment of tendon cells, although it is necessary to consider animal studies to see if similar effects can be obtained in different parts of tendons and ligaments (e.g., shoulder rotator cuff, Achilles tendon, finger tendons, etc.).

## Data Availability

The datasets used and/or analyzed during the current study are available from the corresponding author upon reasonable request.
