# Correction: Ueda et al. Effect of High-Dose Vitamin C on Tendon Cell Degeneration—An In Vitro Study. *Int. J. Mol. Sci.* 2024, *25*, 13358

**DOI:** 10.3390/ijms26189029

**Published:** 2025-09-17

**Authors:** Shusuke Ueda, Toru Ichiseki, Miyako Shimasaki, Daisuke Soma, Masaru Sakurai, Ayumi Kaneuji, Norio Kawahara

**Affiliations:** 1 Department of Orthopaedic Surgery, Kanazawa Medical University, Daigaku 1-1, Uchinada-machi, Kahoku-gun 920-0293, Japan; 2 Division of Translational Research, Department of Life Science, Medical Research Institute, Kanazawa Medical University, Daigaku 1-1, Uchinada-machi, Kahoku-gun 920-0293, Japan; 3 Department of Pathology 2, Kanazawa Medical University, Daigaku 1-1, Uchinada-machi, Kahoku-gun 920-0293, Japan; miya0807@kanazawa-med.ac.jp; 4 Social and Environmental Medicine, Kanazawa Medical University, Daigaku 1-1, Uchinada-machi, Kahoku-gun 920-0293, Japan

In the original publication [1], there was a mistake in Figure 2. An incorrect image was inadvertently used during final preparation. The correct version of Figure 2 appears below. The authors state that the scientific conclusions are unaffected. This correction was approved by the Academic Editor. The original publication has also been updated.

## Figures and Tables

**Figure 2:**
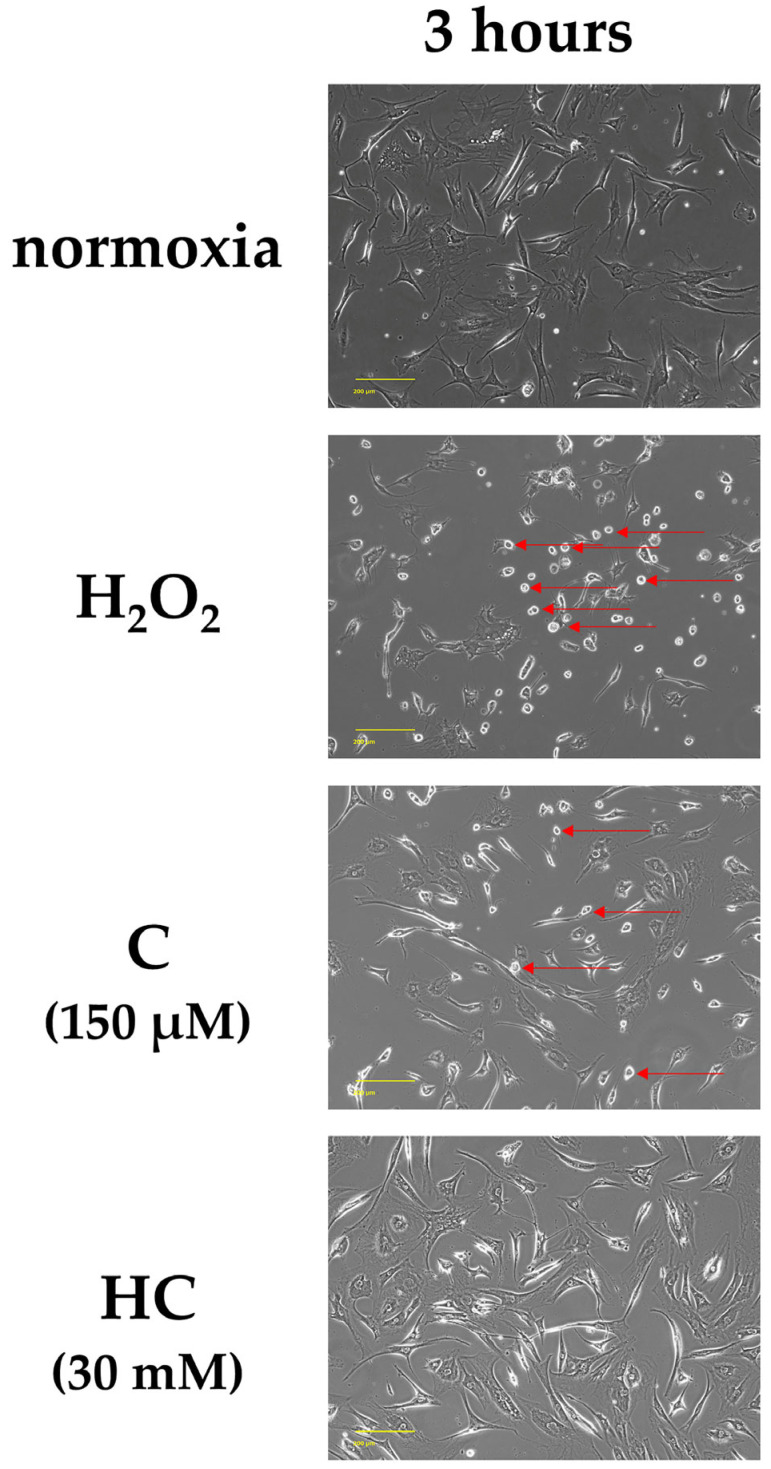
Phase-contrast images of tendon cells at 3 h after H_2_O_2_ exposure. The figures show representative images of various treatment groups. In the normoxia group, most of the cells were engrafted, and almost no detached cells were observed. The H_2_O_2_ group showed a large number of round or shrunken cells. The round and shrunken cells were released, and cell death was observed (red arrows). In the C group, cells with round and shrunken nuclei (red arrows) were mixed with cells that maintained their original forms. Compared with the H_2_O_2_ group, the number of dead cells was reduced in both the C and HC groups. In the HC group, the number of cell deaths was reduced compared to that in the C group. Scale bar: 200 µm.
